# Case Report: A case of advanced renal tuberculosis with recurrent “kidney stones” and specific pathological manifestations

**DOI:** 10.3389/fmed.2026.1809830

**Published:** 2026-05-20

**Authors:** Chongbin Li, Hang Zhang, Ziyi Wang, Yalin Niu, Guiyun Zhu, Jianzhen Liu, Qi Zhao, Wei Li

**Affiliations:** 1 Department of Urology, Hebei Chest Hospital, Shijiazhuang, China; 2 Department of Urology, The Second Hospital of Hebei Medical University, Shijiazhuang, China; 3 Department of Pathology, Hebei Chest Hospital, Shijiazhuang, China

**Keywords:** nephritis, pathology, renal tuberculosis, TB-DNA, Xpert MTB/RIF^®^

## Abstract

**Background:**

Renal tuberculosis is a chronic granulomatous, infectious kidney disease caused by tuberculosis infection, often leading to ureteral tuberculosis, bladder tuberculosis, prostate tuberculosis and genital tract tuberculosis. Its typical pathologic changes are tuberculous nodule formation accompanied by different degrees of caseous necrosis. In recent years, the incidence of atypical renal tuberculosis has increased significantly, with extremely high rates of misdiagnosis and missed diagnosis. Prolonged delays in diagnosis and treatment have led to severe consequences such as renal failure, warranting high priority attention. Even in cases of early-diagnosed renal tuberculosis that achieve “clinical cure” following standardized, full-course anti-tuberculosis drug treatment, some patients still experience severe or even complete renal impairment. Pathological diagnosis can be used to confirm renal tuberculosis. However, what is the pathological outcome after prolonged anti-tuberculosis drug treatment? Does renal tuberculosis complicated by other kidney diseases exhibit specific pathological manifestations? Why does renal function loss occur despite achieving clinical cure? This case report provides answers to these questions.

**Case presentation:**

A 58-year-old female patient with advanced renal tuberculosis was performed nephrectomy ultimately due to loss of renal function even after 4 years of anti-tuberculosis drug treatment. During the treatment for left renal tuberculosis, the patient experienced recurrent left renal calculi and underwent three ureteroscopic lithotripsy procedures along with ureteral stent placement for hydronephrosis drainage. Postoperative pathological changes revealed chronic nephritis, extensive tubular atrophy, glomerulosclerosis, accompanied by renal abscesses and cyst formation. However, given the positive *Mycobacterium tuberculosis* culture 4 years ago, combined with CT imaging findings and a positive T-SPOT.TB test, renal tuberculosis was definitively diagnosed based on positive GeneXpert and TB-DNA PCR results from renal tissue samples, which suggests that *Mycobacterium tuberculosis* persists in the renal tissue despite 4 years of antituberculosis treatment.

**Conclusion:**

Renal tuberculosis complicated by recurrent kidney stones and hydronephrosis, along with suspected urinary tract infection, can lead to atypical pathological changes in renal tissue and exacerbate renal dysfunction. TB-DNA PCR and GeneXpert testing of renal tissue aid in establishing a pathogenetic diagnosis, thereby enhancing the efficacy of pathological diagnosis for renal tuberculosis. Alongside effective, full-course anti-tuberculosis drug therapy and adequate renal pelvic drainage, timely treatment of comorbidities such as renal calculi and urinary tract infections is essential.

## 1 Case presentation

**Figure 1:**
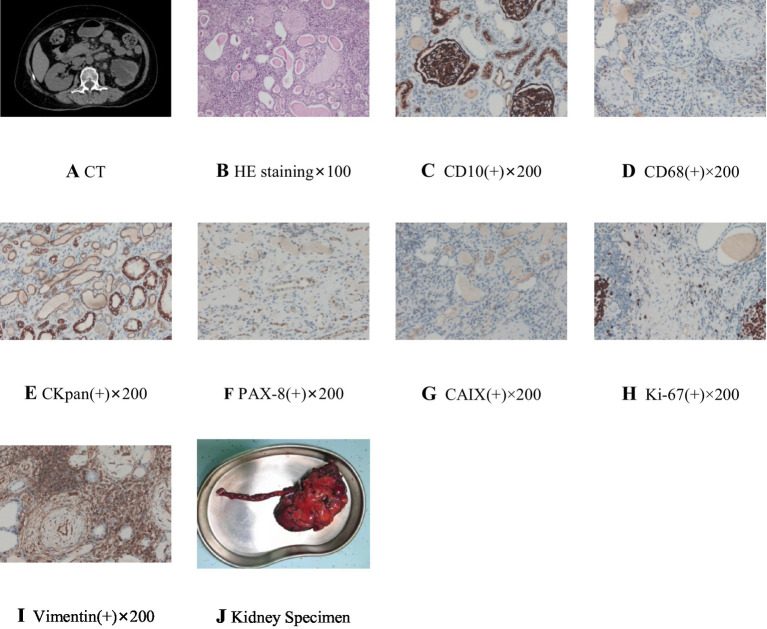
CT and postoperative pathological images. **(A)** CT sacn showed dilated hydronephrosis of the left kidney and upper ureter. **(B)** Postoperative histopathological examination of renal tissue showed that chronic nephritis with extensive tubular atrophy and glomerulosclerosis with renal abscess and cyst formation, HE staining×100. **(C)** CD10 staining is positive in renal tissue, ×200. **(D)** CD68 staining is positive in renal tissue, ×200. **(E)** CKpan staining is positive in renal tissue, ×200. **(F)** PAX-8 staining is positive in renal tissue, ×200. **(G)** CAIX staining is positive in renal tissue, ×200. **(H)** Ki-67 staining is positive in renal tissue, ×200. **(I)** Vimentin staining is positive in renal tissue, ×200. **(J)** The kidney specimen following nephrectomy.

A 58-year-old female patient was diagnosed with “left renal tuberculosis” through *Mycobacterium tuberculosis* (TB) culture and TB-DNA testing 4 years ago at a local hospital and was given anti-tuberculosis treatment for 4 years. The main symptoms include severe frequency, urgency, and dysuria, accompanied by cloudy urine and microscopic hematuria. The initial anti-tuberculosis regimen was HRZE (Isoniazid 0.3 g PO daily, Rifampicin 0.45 g PO daily, Pyrazinamide PO 0.5 g three times daily, and Ethambutol 0.75 g PO daily). After 1 year, due to poor efficacy—which is defined as persistent symptoms of frequent urination, urgency, and dysuria accompanied by weight loss, elevated erythrocyte sedimentation rate, a positive urine TB-DNA, and progression of lesions on CT imaging—the treatment regimen was changed to that Clofazimine 50 mg PO daily, Moxifloxacin 400 mg PO daily, Pasiniazid 0.3 g PO three times daily. And this regimen has been adhered until now, for about 3 years. In addition, within 2 years after the anti-tuberculosis treatment, the patient underwent 3 ureteroscopic lithotripsy operations for recurrent left kidney “stones,” and then ureteral stenting for left hydronephrosis. After the above treatments, the patient had no clinical symptoms. And three consecutive urine sediment tests, including acid-fast staining, *Mycobacterium tuberculosis* culture, PCR TB-DNA, and GeneXpert MTB/RIF, all yielded negative results. Those suggest that this patient has achieved “Clinical Cure” (1) for renal tuberculosis at this stage. But a follow-up urologic CT showed dilated hydronephrosis of the left kidney and upper ureter (Figure 1A), and Renal ECT (Emission Computed Tomography) suggested severe impairment of left kidney function (LGFR 13.8 mL/min, RGFR 64.7 mL/min, GFR, glomerular filtration rate). Besides, blood tests revealed anemia (RBC, red blood cell count was 2.9 × 10^12^/L. Hb, Hemoglobin, was 89 g/L), hypoalbuminemia (Albumin was 25.3 g/L), and accelerated erythrocyte sedimentation rate (ESR was 113 mm/h). Antibodies to *Mycobacterium tuberculosis* (16Kda antibody, 38Kda antibody, LAM antibody, CFP10 antibody, ESAT antibody) and HIV were all negative (−), while T-SPOT.TB (T cell spot detection in tuberculosis infection) was positive (+). Urine analysis showed that pH was 7.0, leukocytes was 3759.40/ul, bacteria was 236/ul, turbidity was positive (+). Urine common bacterial culture was identified as *Proteus mirabilis*, with hyperspectral *β*-lactamase (−) and colony count>10^4^CFU/ml (CFU, colony-forming unit).

Based on the results of drug sensitivity test after bacterial culture, we treated the patient with ceftazidime (1.0 g IV twice daily) for 1 week. The follow-up urine sediment quantitative test results show that WBC (white blood cells) was 3483.4/μl (0–30/μl) and 627.0/HPF (0–5.4/HPF), RBC (red blood cells) was 24.60/μl (0–25/μl) and 4.4/HPF (0–4.5/HPF), bacteria was 78/μl (0–130/μl) and 14.0/HPF (0-24/HPF). Urinalysis results showed leukocytes was strongly positive (++++), turbidity, nitrite and occult blood were all negative (−). The urine test results indicate that the suspected urinary tract infection has been controlled. At the same time, human albumin (20 g IV daily) was given intravenously to correct hypoproteinemia (preoperative Albumin level was 32.9 g/L), and Blood Enrichment Nutri-Geloral (a traditional Chinese medicine combination) to correct anemia (preoperative RBC count was 3.26 × 10^12^/L, Hb was 97 g/L). After perfect preoperative preparation and excluding contraindications to surgery, we performed da Vinci robot-assisted posterior laparoscopic left nephrectomy on the patient. Postoperative pathologic findings showed that chronic nephritis with extensive tubular atrophy and glomerulosclerosis with renal abscess and cyst formation, and chronic inflammation of the ureteral dissection (Figures 1B,J). Immunohistochemistry of renal tissue were performed and showed that CD10, CD68, Ckpan, PAX-8, CAIX, Ki-67 (+) and Vimentin were all positive (+) (Figures 1B–I). And PCR TB-DNA and GeneXpert MTB/RIF assays in kidney tissue were all positive (+). Following nephrectomy, the patient continued standardized treatment with the preoperative anti-tuberculosis regimen for 6 months. After 1 year of follow-up, the patient achieved complete recovery with no recurrence observed (Table 1).

**Table 1:** Summary of diagnosis and treatment process.

Timeline	Diagnosis	Examination methods and results	Treatment	Therapeutic efficacy
4 years ago	Left renal tuberculosis	*Mycobacterium tuberculosis* (TB) culture and TB-DNA testing are all positive.	HRZE was given anti-tuberculosis treatment for 1 years.	Poor efficacy
From 3 years ago to this visiting	Left renal tuberculosis	*Mycobacterium tuberculosis* culture and TB-DNA results were all still positive, and *Mycobacterium tuberculosis* was sensitive to clofazimine, pyrazinamide, and moxifloxacin.	The regime was changed (Clofazimine 50 mg PO daily, Moxifloxacin 400 mg PO daily, Pasiniazid 0.3 g PO three times daily) and that was maintained till this visiting. 3 ureteroscopic lithotripsy operations were performed for recurrent left kidney “stones,” and then ureteral stenting for left hydronephrosis.	Clinical cure
Since this visiting to now	Left renal tuberculosis	CT showed dilated hydronephrosis of the left kidney and upper ureter, and ECT suggested severe impairment of left kidney function. Urinalysis and urine culture indicated urinary tract infection.	Antituberculosis treatment was continued, the urinary tract infection was controlled, anemia and hypoproteinemia were correct, and then left nephrectomy was performed. After the left nephrectomy, the patient continued the preoperative anti-tuberculosis treatment for 6 months before discontinuing the medication.	The renal tuberculosis and recurrent kidney stones were completely cured, and urinary tract infections have not recurred.

## 2 Discussion

In this case report, despite the absence of initial positive urine *Mycobacterium tuberculosis* culture and TB-DNA test results, as well as typical pathological features of renal tuberculosis, the diagnosis of renal tuberculosis was ultimately confirmed based on the patient’s history of effective anti-tuberculosis drug therapy, and positive GeneXpert and TB-DNA PCR test results from the renal tissue following nephrectomy (2–4). After 4 years of anti-tuberculosis treatment, the patient did not have any clinical symptoms of renal tuberculosis such as urinary frequency, urgency, dysuria, and lumbago, and mycobacterium tuberculosis could not be detected by urinary tuberculosis culture, TB-DNA, and GeneXpert (2–4), which confirmed that the anti-tuberculosis regimen was effective. However, there may be the following reasons as to why the pathological changes in the renal tissues of this patient were not typical tuberculous nodules, tuberculous granulomas and caseous necrosis, but showed chronic nephritis-like pathological changes:

Firstly, in the early-stage renal tuberculosis, effective anti-tuberculosis drug treatment has been underwent before the development of typical pathological changes such as tuberculous granulomas and caseous necrosis (5, 6). In this procedure, *Mycobacterium tuberculosis* is rapidly and extensively inactivated or completely eradicated, but the immune response in renal tissue triggered by the infection persists. And this changes ultimately leads to long-term chronic inflammatory pathological damage for renal tissue and ongoing impairment of renal function.

Secondly, recurrent kidney and ureteral stones and urinary tract infections, as well as repeated lithotripsy and stone removal procedures, can indeed cause damage to renal function, which may accelerate renal dysfunction. Although these conditions are not directly related to renal tuberculosis, the inflammation of the renal pelvis and ureter caused by renal tuberculosis serves as a contributing factor to stone formation and concurrent urinary tract infections, and is one of the reasons why the condition is difficult to cure completely (7, 14). The pathological damage caused by stones and urinary tract infections masks the typical pathological manifestation of renal tuberculosis—tuberculous granulomas. A positive result for *Mycobacterium tuberculosis* DNA indicates that the bacteria persist within the renal tissue. This creates a vicious cycle, making the condition difficult to cure and ultimately leading to complete loss of renal function. To conclude, recurrent kidney and ureteral stones combined with hydronephrosis and pyelonephritis lead to chronic inflammation and fibrosis of renal tissue, exacerbating renal dysfunction. These pathological changes mask the inherently atypical features of tuberculous granulomas and focal necrosis (6, 8, 9).

Thirdly, antituberculosis drugs such as rifampicin may also induce allergic interstitial nephritis, which manifests as tubular atrophy and glomerular fibrosis (1).

Fourthly, following prolonged chronic infection and inflammatory repair, tuberculous granulomas undergo organization and progressive fibrosis, ultimately leading to the aforementioned atypical chronic nephritis-like pathological changes and renal dysfunction. This represents the common pathological outcome in most cases of end-stage renal disease. Renal interstitial fibrosis, tubular atrophy and glomerular hyalinosis are frequently observed pathological changes in renal tuberculosis, as confirmed by our previous pathological studies on this condition (6, 8, 10).

Furthermore, this case report also suggests that even after prolonged anti-tuberculosis drug treatment, when clinical symptoms have completely disappeared and clinical cure has been achieved, a minimal latent infection of *Mycobacterium tuberculosis* may still persist in renal tissue (13). TB-DNA PCR and GeneXpert testing demonstrate high sensitivity in the pathological diagnosis of renal tissue. However, dead *Mycobacterium tuberculosis* and its nucleic acid fragments may also yield positive results of nucleic acid test in renal tissue. This can be confirmed through TB-RNA testing and *Mycobacterium tuberculosis* culture of tissue specimens. Unfortunately, we did not perform these tests at that time.

The pathological findings of this case of renal tuberculosis reveal extensive tubular atrophy and glomerular sclerosis in the renal tissue, which can cause direct damage to the kidney’s filtration and reabsorption functions. Focal abscesses and small cysts in the kidney, along with immunohistochemical staining such as CD10(+) and CD68(+), suggest focal infection and chronic inflammation of renal tissue (11, 12). PAX-8(+) is a normal expression in renal tissue cells. CKpan(+) indicates positive cytokeratin expression and reactive proliferation of epithelial cells. CAIX(+) indicates positive carbonic anhydrase IX expression and tissue hypoxia. Ki-67(+) indicates active inflammatory cell proliferation. These findings suggest the presence of active, long-term chronic inflammatory damage and tissue repair in the renal tissue (11, 12). Based on the medical history, imaging studies, and pathological examination, renal tumors can be ruled out. Vimentin (+) indicates reactive proliferation of fibroblasts within renal tissue and extensive fibrosis of the renal interstitium (11, 12). This can lead to compression and fibrosis of renal interstitial vessels, resulting in pre-renal renal tissue ischemia and subsequent pre-renal renal dysfunction. Chronic ureteral inflammation can cause ureteral stricture, leading to secondary hydronephrosis and ureteral dilatation, resulting in post-renal obstructive renal dysfunction.

In conclusion, renal tuberculosis complicated by recurrent kidney stones and hydronephrosis, along with suspected urinary tract infection, leads to atypical pathological changes in renal tissue and exacerbate renal dysfunction. It serves as a warning that pathological changes in renal tuberculosis can be highly atypical. Even after extended anti-tuberculosis drug treatment, latent *Mycobacterium tuberculosis* infection may persist in renal tissue. TB-DNA PCR and GeneXpert testing of renal tissue aid in establishing a pathogenetic diagnosis, thereby enhancing the efficacy of pathological diagnosis for renal tuberculosis. Alongside effective, full-course anti-tuberculosis drug therapy and adequate renal pelvic drainage, timely treatment of comorbidities such as renal calculi and urinary tract infections is essential.

## Data Availability

The original contributions presented in the study are included in the article/supplementary material, further inquiries can be directed to the corresponding authors.

## Glossary

MTB/TB: *Mycobacterium tuberculosis*
DNA: deoxyribonucleic acid
PCR: Polymerase Chain Reaction
GeneXpert MTB/RIF: rifampicin resistant real-time fluorescent quantitative nucleic acid amplification
HRZE: Isoniazid, Rifampicin, Pyrazinamide, and Ethambutol
SPOT.TB: T cell spot detection in tuberculosis infection
HE: hematoxylin eosin
PO: per os
IV: intravenous drip
CT: Computed Tomography
ECT: Emission Computed Tomography
LGFR: Left Glomerular Filtration Rate
RGFR: Right Glomerular Filtration Rate
ESR: erythrocyte sedimentation rate
LAM: Lipoarabinomannan
CFP10: Culture Filtrate Protein 10
ESAT: Early Secreted Antigenic Target 6
HIV: Human Immunodeficiency Virus
CD10: Cluster of Differentiation 10
Ckpan: Cytokeratin Pan
PAX-8: Paired box gene 8
CAIX: carbonic anhydrase IX
Ki-67: nuclear-associated antigen ki-67
